# Omega-3 Biotechnology: A Green and Sustainable Process for Omega-3 Fatty Acids Production

**DOI:** 10.3389/fbioe.2015.00158

**Published:** 2015-10-12

**Authors:** Xiao-Jun Ji, Lu-Jing Ren, He Huang

**Affiliations:** ^1^State Key Laboratory of Materials-Oriented Chemical Engineering, College of Biotechnology and Pharmaceutical Engineering, Nanjing Tech University, Nanjing, China

**Keywords:** algae, docosahexaenoic acid, eicosapentaenoic acid, omega-3 fatty acid, triglyceride

Omega-3 fatty acids are known as essential fatty acids because they are important for good health. They have many positive effects on human beings, such as anti-inflammatory and anti-blood clotting actions, lowering triglyceride (TAG) level, reducing blood pressure, and reducing the risks of diabetes, some cancers, etc. (Wen and Chen, 2003; Ren et al., 2010; Xie et al., 2015). The human body cannot synthesize these fatty acids on its own. Therefore, the omega-3 fatty acids must be obtained from the diet.

Eicosapentaenoic acid (EPA, C20:5, n-3) and docosahexaenoic acid (DHA, C22:6, n-3) are two typical omega-3 fatty acids. Their traditional source is derived from cold-water fish oils. However, mass-scale fisheries are not much longer sustainable if we continue on the growing demand for these fish products. Reliance on the fish oil as the source of omega-3 fatty acids is also complicated by the significant taste, odor, and stability problems associated with this type of oil. Furthermore, product quality derived from fish oil is generally dependent on the season and location, and it can be affected by the ocean pollution. The process for purifying these fatty acids from fish oil itself is complicated as well (Lenihan-Geels et al., 2013). All these complications limit the use of fish oil as a food additive or food supplement. Alternatively, novel sources of omega-3 fatty acids can be green manufactured from marine algal or algae-like microbial oils, which could eliminate many of the taste and odor problems associated with fish and discard the shortcomings of fish oil-based process. The process of culturing the algae or algae-like microorganism to accumulate the oil rich in omega-3 fatty acids was defined as “Omega-3 Biotechnology” (Gupta et al., 2012).

Currently, the most common algae or algae-like microorganism used for the production of DHA belong to the marine members of the families Thraustochytriaceae and Crypthecodiniaceae. The Thraustochytrids include the genera *Schizochytrium* and *Ulkenia*, whereas dinoflagellate *Crypthecodinium* is a genus of the family Crypthecodiniaceae (Barclay et al., 1994; Borowitzka, 2013; Klok et al., 2014). Members of these genera are widely dispersed in the oceans of the world. By heterotrophically culturing these microorganisms, the omega-3 biotechnological processes for DHA production have gone into industrial scale (Ren et al., 2010). However, the production of EPA is still being restricted to laboratory scale. The traditionally used EPA producers are the algae *Phaeodactylum tricornutum*, *Nannochloropsis*, and *Nitzchia* (Wen and Chen, 2003). The relatively low accumulated biomass and slow growth rate of these algae hindered the industrial EPA production. Recently, the metabolically engineered yeast, *Yarrowia lipolytica*, has been used to commercially produce EPA in an industrial mass scale by the E.I. DuPont Company (Xie et al., 2015).

Apart from the algal or microbial fermentation procedure, the omega-3 biotechnological process also includes efficient and green downstream procedures, i.e., oil extraction and refining process. The oil product rich in omega-3 fatty acid obtained is a kind of intracellular metabolite; therefore, before extracting the oil from the algal (microbial) biomass, the cells must be disrupted first. Traditionally, the method used was mechanical based, and its high energy requirements pose a major challenge. Alternatively, the novel enzymatic disruption method has been developed to strengthen the process economic efficiency, as it costs lower energy and increases the efficiency of the following extraction process. The enzymatic disrupted cell biomass is blended with hexane in a continuous extractor. Afterward, the mixture is pumped into a separator and then fed into the desolventizer to obtain the crude oil product. However, the composition of extracted crude oil is complex (Armenta and Valentine, 2013). Impurities, mainly phospholipids and other polar lipids, and any volatile materials that may adversely affect the smell or taste of the oil, must be removed prior to be used as high-value nutrient. They can be removed to obtain the TAG components by using the oil refinement and deodorization procedures. These processes are exactly the same as those used in conjunction with other plant oils and do not need any major changes in their technology to manage the oil product. Because of the sensitivity of oil rich in omega-3 fatty acids to oxidative damage, the best processing operations use short reaction times at reduced temperatures and with a constant blanket of nitrogen throughout the process. The oil is bleached, filtered, and deodorized with a thin-film continuous deodorizer to the final product of clear, yellow TAG oil, with specified limits for unsaponifiables and free fatty acids.

In summary, the omega-3 biotechnological process is a green and sustainable process for the omega-3 fatty acid production. In the future, exploration for diverse algae species or microbe isolates having fast growth rates, high biomass content, and high oil accumulating capability will further enhance the efficiency of omega-3 biotechnological process. At the same time, with the help of numerous novel research tools and more integrative information based upon studies in genomics, proteomics, metabolomics, and systems biology, algae or microbe strains would not only achieve higher omega-3 fatty acid percentages in the oil product but would also obtain higher omega-3 fatty acid-rich oil productivity. Furthermore, one of the major challenges to omega-3 biotechnological process will be the development of an efficient and economical downstream process with fewer steps and lower level of solvents.

## Conflict of Interest Statement

The authors declare that the research was conducted in the absence of any commercial or financial relationships that could be construed as a potential conflict of interest.
